# High-Resolution X-Ray Structure of the Trimeric Scar/WAVE-Complex Precursor Brk1

**DOI:** 10.1371/journal.pone.0021327

**Published:** 2011-06-20

**Authors:** Joern Linkner, Gregor Witte, Theresia Stradal, Ute Curth, Jan Faix

**Affiliations:** 1 Institute for Biophysical Chemistry, Hannover Medical School, Hannover, Germany; 2 Gene Center and Department of Biochemistry, Munich Center for Advanced Photonics (MAP) and Center for Integrated Protein Science Munich (CIPSM) at the Ludwig-Maximilians-University, Munich, Germany; 3 Institute for Molecular Cell Biology, University of Muenster, Muenster, Germany; 4 Signaling and Motility Group, Helmholtz Centre for Infection Research (HZI), Braunschweig, Germany; Institute of Molecular and Cell Biology, Singapore

## Abstract

The Scar/WAVE-complex links upstream Rho-GTPase signaling to the activation of the conserved Arp2/3-complex. Scar/WAVE-induced and Arp2/3-complex-mediated actin nucleation is crucial for actin assembly in protruding lamellipodia to drive cell migration. The heteropentameric Scar/WAVE-complex is composed of Scar/WAVE, Abi, Nap, Pir and a small polypeptide Brk1/HSPC300, and recent work suggested that free Brk1 serves as a homooligomeric precursor in the assembly of this complex. Here we characterized the Brk1 trimer from *Dictyostelium* by analytical ultracentrifugation and gelfiltration. We show for the first time its dissociation at concentrations in the nanomolar range as well as an exchange of subunits within different DdBrk1 containing complexes. Moreover, we determined the three-dimensional structure of DdBrk1 at 1.5 Å resolution by X-ray crystallography. Three chains of DdBrk1 are associated with each other forming a parallel triple coiled-coil bundle. Notably, this structure is highly similar to the heterotrimeric α-helical bundle of HSPC300/WAVE1/Abi2 within the human Scar/WAVE-complex. This finding, together with the fact that Brk1 is collectively sandwiched by the remaining subunits and also constitutes the main subunit connecting the triple-coil domain of the HSPC300/WAVE1/Abi2/ heterotrimer to Sra1(Pir1), implies a critical function of this subunit in the assembly process of the entire Scar/WAVE-complex.

## Introduction

Cells harness the power of actin polymerization for the formation of protruding membrane sheets filled with a dense actin filament network at the leading edge referred to as lamellipodia (or pseudopodia in *Dictyostelium*) to drive cell migration [1]–[3]. According to the current knowledge, actin nucleation in lamellipodia and ruffles is accomplished by the Arp2/3-complex [4]–[6]. However, since purified Arp2/3-complex is intrinsically inactive [7], [8], it requires activation by so-called ‘nucleation promoting factors’ (NPFs), such as WASP or WAVE proteins [9]–[12]. The first member of WAVE family proteins was initially identified in *Dictyostelium* as a suppressor of a cyclic AMP receptor mutant and was for that reason named Scar [13]. The Scar/WAVE NPFs are required for plasma membrane projections in diverse processes such as lamellipodia formation in migrating animal cells [14], [15], dendritic spine morphology in neurons [16] or trichome morphogenesis in plant cells [17], [18]. Genetic inactivation of *WAVE* genes in the mouse or in several commonly used cell lines severely impedes the formation of lamellipodia [5], [14], [15], [19], corroborating their critical role in the activation of the Arp2/3-complex during cell migration. In contrast to WASP-proteins, which remain inactive by intramolecular autoinhibition until activation by Rho GTPases, isolated Scar/WAVE proteins are fully active outside the Scar/WAVE-complex [20], [21]. The Scar/WAVE subunit is kept inactive by various interactions within the Scar/WAVE-complex, which consists of the five subunits Nap/Hem, Pir/Sra/CyFip, Abi, Scar/WAVE and Brk1/HSPC300, in a 1∶1∶1∶1∶1 stoichiometry [21]–[23]. The Scar/WAVE-complex has been recently reported to be activated by multiple factors including active Rac and acidic phospholipids, by releasing the C-terminal VCA domain of Scar/WAVE to activate the Arp2/3-complex, and linking upstream Rho-family GTPase signaling to the activation of the Arp2/3-complex in different organisms [21], [23]–[25]. Recent exciting work reporting on the structure of the human heteropentameric Scar/WAVE-complex, revealed details of its inactive state and how Rac binding could lead to the release of the masked VCA domain, hence activating the Scar/WAVE-complex [23]. Moreover, and contrary to previous assumptions, Brk1 instead of Abi is forming the core subunit of the complex [23]. Despite considerable knowledge about activation of the Scar/WAVE-complex, its assembly process remains elusive. In order to further our knowledge, of how the Scar/WAVE-complex is assembled, it is instrumental to obtain structural information of precursor and intermediate subcomplexes. Interestingly, in vertebrates, Brk1 forms homooligomers that remain stable as a free subcomplex in the absence of other Scar/WAVE-complex subunits [22], [26]. This is remarkable, as the depletion of one subunit commonly leads to degradation of at least the Scar/WAVE and Abi proteins [26]–[30]. After depletion of Brk1 in mammalian and *Dictyostelium* cells Scar/WAVE proteins are almost undetectable, whereas the level of PirA in *Dictyostelium* Brk1-null, Scar-null and AbiA-null mutants seems nearly unaffected [31], [32]. However, depletion of NapA in *Dictyostelium* caused a marked reduction of PirA [31]. Expression of tagged or untagged Brk1 in *Dictyostelium* Brk1-null cells restores Scar protein levels almost completely, pointing out that Brk1 is required for stability of Scar/WAVE proteins [26], [32]. Notably, electroporation of recombinant oligomeric Brk1 into Brk1-depleted HeLa S cells not only restored protein levels of other Scar/WAVE-complex components, but also incorporated into the heteropentameric Scar/WAVE-complex [26], suggesting its potential role as a precursor of the Scar/WAVE-complex [33]. *Dictyostelium* Brk1 was first identified as an ortholog of human HSPC300 and was accordingly named DdHSPC300 [32]. However, since human HSPC300 apparently originates from an erroneously annotated cDNA, corresponding to human Brk1, carrying a point mutation in its stop codon and therefore encompassing 35 extra amino acid residues, the *Dictyostelium* protein is more closely related to human Brk1. Thus, herein we refer to the proteins as DdBrk1 and HsBrk1, respectively. Here we present the high resolution structure and a biochemical analysis of DdBrk1, which provides new insights on the question of how Brk1 may function as a precursor during the assembly of the mature Scar/WAVE-complex.

## Results and Discussion

### Brk1 is stable outside the functional Scar/WAVE-complex

**Figure 1:**
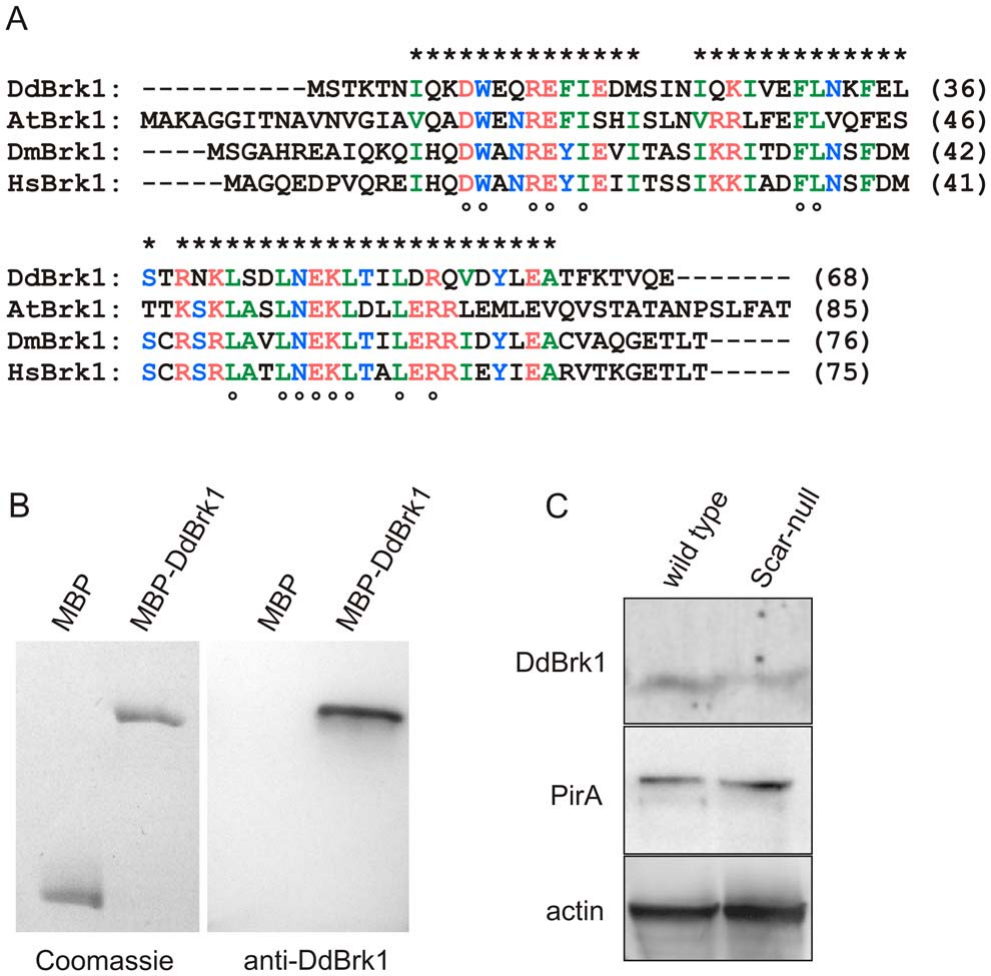
DdBrk1 and PirA remain stable in the absence of Scar. (A) DdBrk1 is evolutionary conserved. Multiple sequence alignment of Brk1 from different species using the MUSCA algorithm [56]. Black letters show non-similar residues; letters in identical color display amino acids with similar hydropathy; stars on top depict blocks of conserved amino acids and open circles below indicate identical residues. At: *Arabidopsis thaliana* (NP_179849); Dd: *Dictyostelium discoideum* (XP_641829); Dm: *Drosophila melanogaster* (NP_726400); Hs: *Homo sapiens* (AAF28978). (B) The specificity of anti-DdBrk1 polyclonal antibodies was assessed by Western blotting. (Left) MBP and MBP-DdBrk1 were separated by a 10% SDS-PAGE and visualized by Coomassie-blue stain. (Right) Immunoblot analysis of the same proteins after transfer onto a PVDF membrane and detection with anti-DdBrk1 antibodies. The antibodies specifically detected the DdBrk1 containing sample, but did not bind to the MBP moiety, which served as a negative control. (C) DdBrk1 and PirA remain stable in Scar-null cells. Total cellular proteins corresponding to 2×10^5^ wild-type or Scar-null cells were separated by SDS-PAGE using 16% Tris-Tricine or 10% Tris-Glycine gels, transferred to a PVDF membrane and labelled by anti-DdBrk1 antibodies. The same samples were also probed with anti-PirA antibodies. The Western blot with anti-actin antibody mAb 224–236–1 [45] shows equal sample loading. Densitometric analysis of stained bands revealed that DdBrk1 was moderately reduced while the PirA level was unchanged in the absence of Scar.

Brk1 is important for the stability of the Scar/WAVE subunit in different organisms [26], [32], [34], [35], and was proposed to function as a precursor for Scar/WAVE-complex assembly in Hela S cells [26]. *Dictyostelium* DdBrk1 encompasses 68 residues and shares 37% sequence identity and 52% similarity with human Brk1/HSPC300 (HsBrk1) (Fig. 1A). To test whether DdBrk1 remains stable without functional Scar/WAVE-complex and therefore could potentially function as a precursor of the Scar/WAVE-complex in *D. discoideum*, we raised antibodies against recombinant DdBrk1 (Fig. 1B), and compared the protein levels in *Dictyostelium* wild-type and Scar-null cells by Western-blotting. Analysis of the stained bands revealed that the DdBrk1 level in the Scar-null mutant was reduced by about 40% when compared to wild-type cells (Fig. 1C). In agreement with previous results, the PirA subunit also remained stable in the absence of Scar and/or Abi [32], [36], supportive of mutual stabilization of Nap and Pir proteins by heterodimerization [22], [23]. Consistently, Pir/Sra is instable after elimination or depletion of Nap in different systems and cell lines [5], [31], [37]. Taken together, these findings suggest that Brk1 is the subunit of the Scar/WAVE-complex that shows least pronounced degradation in the absence of any other subunit [26], and can abundantly persist in the absence of any other subunit of this complex. Notably, in DdBrk1-null cells Scar is undetectable, however, its level could be fully restored by the expression of either recombinant tagged or untagged DdBrk1 [32]. Therefore DdBrk1 potentially acts as a precursor of the Scar/WAVE-complex in *Dictyostelium* cells. This assumption is further supported by the fact that, in addition to the Scar/WAVE-complex, an excess of Brk1 is found in the cytosol of HeLa S cells [22].

### DdBrk1 forms homotrimers in solution

**Figure 2:**
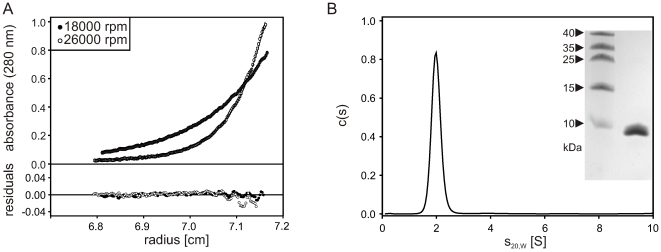
DdBrk1 forms stable trimers in solution. Analytical ultracentrifugation experiments of 43 µM DdBrk1 in PBS at a detection wavelength of 280 nm. (A) Sedimentation equilibrium gradients were measured at rotor speeds of 18,000 rpm and 26,000 rpm at 10°C. Global fitting of the data with a model of a single species using the program BPCfit [46] yielded a molar mass of 24.7 (±2) kg/mol (solid lines) indicating that the protein forms trimers in solution. (B) Sedimentation coefficient distribution as obtained from a sedimentation velocity experiment at 20°C using the program SEDFIT [48]. DdBrk1 sediments as a single species with a sedimentation coefficient s_20,W_ = 2.1 S. Experiments performed in a concentration rage of 15 µM to 340 µM DdBrk1 gave no indication of aggregation or dissociation of the DdBrk1 trimers, as indicated by a slight decrease of the sedimentation coefficient with increasing protein concentration (data not shown). The inset shows a Coomassie stained 16%-Tris/Tricin SDS-PAGE of the DdBrk1 sample used in these experiments.

In order to further characterize DdBrk1, we expressed it as a GST-fusion protein in *E. coli*. After removal of the GST-tag and additional purification (see Material and Methods) DdBrk1 was subjected to sedimentation equilibrium experiments in the analytical ultracentrifuge to determine the molar mass in solution. Equillibrium concentration gradients of 43 µM and 171 µM DdBrk1 obtained at two different rotor speeds could be globally fitted with a single molar mass of 24.7 (±2) kg/mol (for 43 µM, see Fig. 2A). Since the molar mass of the protein calculated from its amino acid composition is 8.61 kg/mol, these data clearly show that under these conditions DdBrk1 forms homotrimers in solution.

To further characterize its hydrodynamic properties, DdBrk1 was examined in sedimentation velocity experiments in a concentration range of 15 µM to 340 µM. The c(s) distributions showed a single species with s_20,W_ = 2.1 S (for an example see Fig. 2B), and no indication of aggregation or dissociation in the concentration range used. As expected for a system that does not change its oligomerization state with protein concentration, s_20,W_ was slightly decreasing with increasing protein concentration (data not shown) [38]. Therefore, DdBrk1 forms stable trimers at concentrations in the micromolar range. From the molar mass of the homotrimer and the obtained sedimentation coefficient a frictional ratio f/f0 = 1.5 and a hydrodynamic radius r_H_ = 2.9 nm were calculated. Since for hydrated spherical proteins f/f0 is expected to be in the range of 1.1 to 1.2 [39], the shape of the DdBrk1 trimer appears to deviate substantially from a sphere. This suggests an elongated shape and/or the presence of unstructured loops.

### Crystal structure of DdBrk1

**Figure 3:**
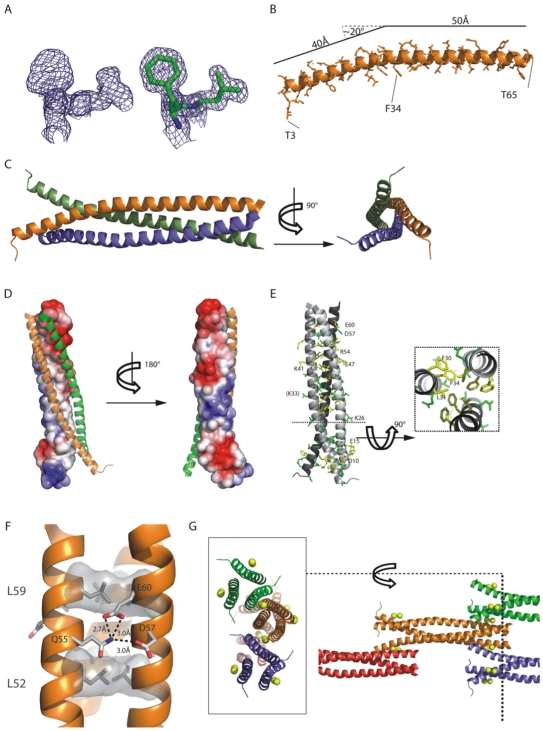
X-ray Structure of DdBrk1. (A) Portions of experimental (left) and final refined 2fo-fc electron density shown with residues (right) of the region F30-L31 contoured at 1σ. (B) Cartoon model of the completely α-helical DdBrk1-molecule in the asymmetric unit. The approximately 20° kink at K-33 creates the N-terminally funnel shape in the trimer. (C) Two orthogonal views of the trimeric DdBrk1, built up from symmetry mates from the 3-fold symmetry axis. Whereas the C-termini are tightly packed, the N-termini seem to open up and create a funnel-like shape. (D) Solvent accessible electrostatic surface potential of one DdBrk1-chain (ranging from blue = 3kT/e to red = -3kT/e) calculated with APBS/PyMol [57]. The other two chains of the trimer are shown as cartoon representations in green and orange. The helix-helix-interaction surface is neutral whereas the outside of the supercoil is clearly hydrophilic and shows negatively charged patches at the N- and C-termini and a positive patch in the central part. (E) Most of the highly conserved residues are located in the hydrophobic interface (e.g. cross-section with F30, L31, F34), but charged residues on the surface-patches are also highly conserved. Identical residues depicted in Figure 1A are shown in yellow and highly conserved residues are shown in green. (F) Section of the triple-coil showing a typical leucine heptad-repeat (L52, L59, shaded in grey) and the ring-like stabilization between Q55 and D57/E60 (shown as sticks) of the neighboring chain. (G) The crystal lattice of DdBrk1-crystals is stabilized by two Ca^2+^-ions in a head-to-tail orientation of the Brk1-chains. The two ions are coordinated by one N-terminal charged patch (W11, E15, E18)_chain 1_ and two C-terminal patches from two chains (D53, D57, E60)_chain 2_ and (Q55)_chain 3_. The picture depicts an exemplary part of the crystal lattice (right part, some molecules were omitted for clarity) and a cross-section (left part) to visualize the coordinated Ca^2+^-ions (yellow spheres). DdBrk1 chains are shown as cartoon representations and colored as biological units.

To learn more about the structure of the trimeric DdBrk1-complex we crystallized DdBrk1 and determined the structure by X-ray crystallography. As DdBrk1 does not contain any methionines that could be used for selenomethionine substitution, and no molecular replacement model was available, we co-crystallized DdBrk1 in presence of heavy atom salts and collected a 1.5 Å resolution data set of platinum soaked crystals at Pt-peak wavelength. Phasing using SHELX [40] yielded two well defined anomalous sites and well-interpretable electron density for the protein chain (Fig. 3A). The first automatically built model [41] obtained from this anomalous dataset was used to phase a high-multiplicity native dataset which then was used in further refinement steps. However, during the refinement process it turned out that the native crystal contained two anomalous sites, indicating that these sites were possibly not occupied by platinum in the soaked crystals. As DdBrk1 was crystallized in presence of 0.2 M Ca^2+^-ions, it is very likely that the anomalous signal of calcium at platinum peak-wavelength was sufficient for phasing of the relatively small protein due to the high resolution data and high multiplicity of the measurement. Thus, the refinement of the dataset of the native crystal was performed including two Ca^2+^-ions (Table 1). The final model depicted in Figure 3A–C containing one DdBrk1-molecule in the asymmetric unit (residues 3–65), two molecules of MPD, two Ca^2+^-ions and 107 solvent molecules was refined to an R-value of 0.159 (R_free_ = 0.182) and showed very good geometry.

**Table 1:** Data collection, phasing and refinement statistics.

	Native	Anomalous
Beamline	ESRF ID23-2	SLS-X06DA
Spacegroup	P6_3_	P6_3_
Cell dimensions (Å)	39.14, 39.14, 118.19	38.89, 38.89, 117.76
		
Wavelength (Å)	0.8726	1.0722
Resolution (Å)	20–1.5	50–1.5
Rmeas (%)	5.2 (28.1)	4.1 (55.7)
I/σ	30.35 (8.95)	24.46 (3.07)
Completeness	98.6 (97.4)	98.9 (97.3)
Multiplicity	12.49 (12.09)	4.76 (4.62)
		
**Refinement**		
Resolution (Å)	20–1.5	
Number of unique reflections	16152	
Rwork/Rfree (%)	15.89/18.17	
Protein non-hydrogen atoms	595 (DdBrk1 residues 3–65)	
MPD	16 (2 molecules of MPD)	
Ca^2+^	2	
Water	107	
B-factors (average)		
Protein	22.74	
Ca^2+^	9.69	
MPD	38.18	
Water	39.87	
RMSD		
Bond lengths (Å)	0.013	
Bond angles (°)	1.245	
PDB code	3PP5	

Values in parentheses are for highest resolution shell.

*Rfree is calculated for a randomly chosen 5% of reflections.

The DdBrk1-chain forms a single continuous α-helix (Fig. 3B) and assembles into a triple-coiled-coil (left handed superhelix) with its symmetry molecules (Fig. 3C) along a conserved hydrophobic patch (Fig. 3D–E). This triple-helix is stabilized via typical knobs-into-holes interactions with heptad-repeats of lysine residues [42] with an interface area of 935Å^2^ between the single helices. Hydrogen bonds between Q55 of one chain and D57/E60 in the neighboring chain further stabilize the C-terminal triple-coil domain like a ring (Fig. 3F), as also observed in the structure of the actin-binding protein coronin 1 [43]. As the helix shows an approx. 20° bend at K33, residues 3–32 are opening up the triple-coil to form a funnel shaped N-terminal region. The entry into the triple-coil structure is highly hydrophobic and occupied by larger hydrophobic residues with additional stacking interactions (F30, F34). Residues 33–65 fold into a compact triple-coil structure whereas the missing residues of the N-terminus are probably flexible and thus can not be seen in the electron density.

**Figure 4:**
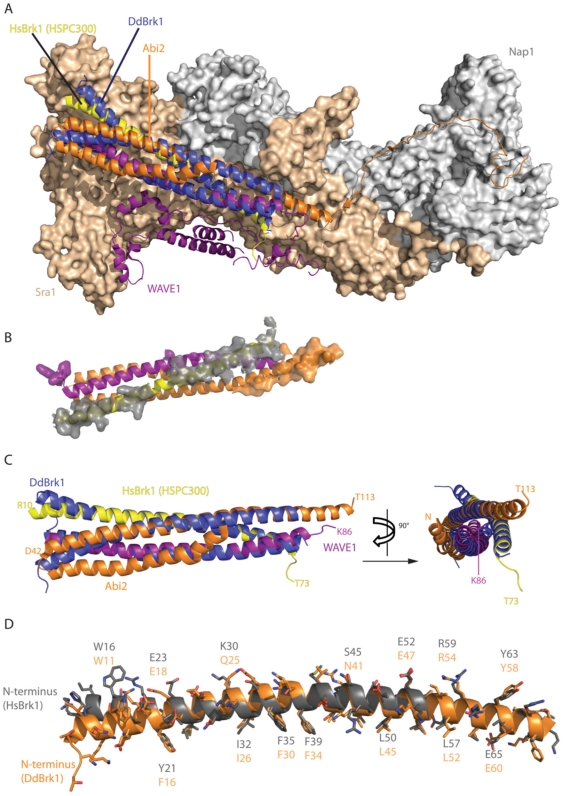
Comparison of DdBrk1 with human WAVE-complex. (A) Superimposition of homotrimeric DdBrk1 with the human hetero-pentameric Scar/WAVE-complex [23] (pdb code: 3P8C). The subunits HSPC300/HsBrk1 (yellow), WAVE1 (purple) and Abi2 (orange) are shown as cartoon representations using the same color code as in [23], Nap1 and Sra1 (also known as Pir1) are shown as surfaces in grey and beige. The DdBrk1-triple helix (blue cartoon) was superimposed onto the HsBrk1/WAVE1/Abi2-subcomplex to illustrate the structural similarity between homotrimeric DdBrk1 and the heterotrimeric subcomplex within the mature Scar/WAVE-complex (RMSD = 1.055Å). (B) Contact surface of the human triple-coil assembly to the Nap1/Sra1-platform as shown in (A). The triple-coil assembly is shown from the binding interface side, obtained by rotation of 180° around the horizontal axis. Residues within 5Å of the platform are shown with their surfaces (for reason of clarity the surface of HsBrk1-contact residues is shown in transparent grey to enhance contrast). HsBrk1 is responsible for the majority of contacts between the triple-coil arrangement and the Nap1/Sra1-platform. (C) Isolated superimposition of the triple-coil domains from the heterotrimer HsBrk1/WAVE1/Abi2/ (purple, orange, yellow) and the DdBrk1 homotrimer (blue) in two orthogonal projections. (D) A detailed superposition with exemplary labeled side chains of HsBrk1 (grey) and DdBrk1 (orange) underlining the high similarity both in sequence and structure of the two adapter-proteins.

As the crystal packing showed two Ca^2+^-ions coordinated by three chains in the head-to-tail arrangement in the lattice (Fig. 3G), also involving residues of the stabilizing ring at the C-terminus of DdBrk1 (Q55, D57/E60), we also tested whether ions may play a role in oligomerization behavior of DdBrk1-trimers. For this reason we examined DdBrk1 in the presence or absence of Ca^2+^-ions by sedimentation velocity experiments in the analytical ultracentrifuge. However, under all conditions examined, DdBrk1 formed a stable trimer independent of the Ca^2+^-concentration (data not shown), indicating that this interaction is more likely a crystallographic phenomenon. This is consistent with the recently solved structure of human Scar/WAVE-complex [23], since neither Ca^2+^-ions nor chelating side chains in the neighboring subunits are present. A more detailed analysis of this heteropentameric complex with focus on the triple-coil domains of the HsBrk1(HSPC300)/WAVE1/Abi2-subcomplex (pdb code: 3P8C) suggests that Sra1(Pir1) largely serves as a binding platform for the heterotrimeric triple-coil complex, with additional contacts between the C-terminus of Abi2 and Nap1. The superposition of homotrimeric *Dictyostelium* DdBrk1-structure with the triple-coil arrangement of human Brk1/WAVE1/Abi2-subunits shows a reasonable good fit (Fig. 4). The interaction of the hetero-trimeric triple coiled-coil domain with the platform Sra1 is mainly mediated by HsBrk1 (Fig. 4B). As HsBrk1 and DdBrk1 are highly homologous in sequence (Fig. 1) and structure (Fig. 4D), we propose a similar arrangement of the complex in *Dictyostelium*. The α-helical regions within WAVE1 (helix 1) and Abi2 (helix 2) interacting with the human Brk1 subunit have a very similar length when compared with the DdBrk1 subunit (Fig. 4C). Coiled-coil regions of similar length were also predicted for AbiA and Scar from *Dictyostelium* using PCOIL (http://toolkit.tuebingen.mpg.de/pcoils, data not shown), indicating a conserved mechanism of the assembly process even across evolutionary distinct species. The fact that Brk1 proteins are found only in one copy in the pentameric Scar/WAVE-complex raised the interesting question of how Brk1, Scar/WAVE and Abi proteins assemble into a heterotrimeric subcomplex, which must be accompanied with breaking up symmetry of the homotrimeric DdBrk1 assembly observed in our crystal structure. In the structure of the human complex (pdb code: 3P8C) WAVE1 is additionally binding to Sra1 via its C-terminal domain and Abi2 is contacting the surface of Nap1 via its C-terminus. These additional contacts might very well support heterotrimer formation as they introduce asymmetry and contribute to the binding energy, and would be not possible if a homotrimeric Brk1 binds to the Sra1/Nap1 platform.

### Trimeric DdBrk1 is in dynamic equilibrium with its monomeric form

**Figure 5:**
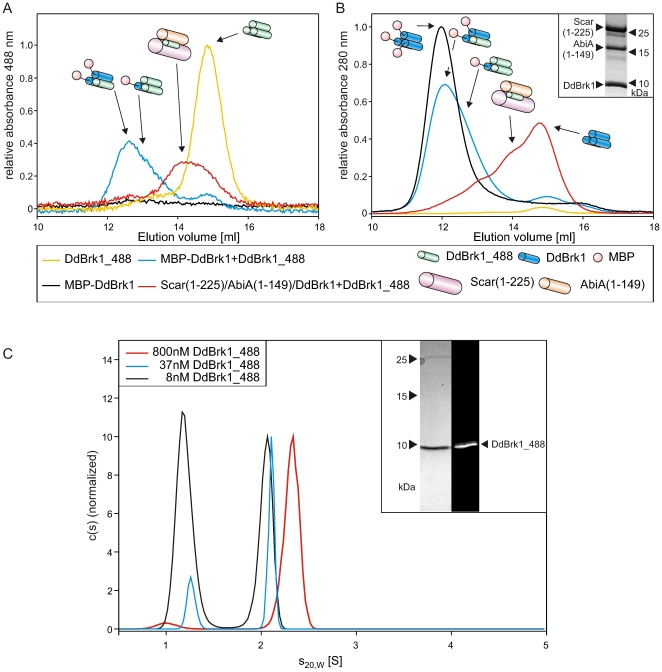
DdBrk1 chains swap within DdBrk1-containing complexes. MBP-DdBrk1 homotrimers and Scar(1-225)/AbiA(1-149)/DdBrk1-complexes were incubated with DdBrk1_488 and subjected to gelfiltration. (A) The elution of 200 nM DdBrk1_488 alone and 100 nM DdBrk1_488 after incubation with other DdBrk1 containing complexes was monitored at 488 nm. 100 nM DdBrk1_488 showed an increased r_H_ in the presence of 1.4 µM MBP-DdBrk1- and Scar(1–225)/AbiA(1–149)/DdBrk1-complexes, confirming an exchange of subunits within these complexes. The unlabeled complexes were undetectable in the absence of DdBrk1_488 at 488 nm absorption. (B) Simultaneously recorded elution profiles at 280 nm showed slightly decreased hydrodynamic radii of the MBP-DdBrk1 fusion construct after incubation with DdBrk1_488, indicating different stoichiometries of trimeric MBP-DdBrk1/DdBrk1_488-complexes. The inset shows a 16% Tris-Tricine SDS-PAGE stained with Coomassie blue of copurified Scar(1–225)/AbiA(1–149)/DdBrk1 used in these experiments. For reasons of clarity, the different complexes are schematically shown in addition to the elution profiles. (C) Sedimentation velocity experiments of DdBrk1_488 showed dissociation of the homotrimer in the lower nanomolar range. The decreasing s-value of the faster sedimenting boundary at decreasing protein concentrations indicates a fast dissociation of the homotrimer when compared to the time scale of the experiment. The inset shows a 16% Tris-Tricine SDS-PAGE of DdBrk1_488 visualized by Coomassie-blue stain and ultraviolet illumination.

To address the issues, whether DdBrk1 homotrimers dissociate at nanomolar protein concentrations, exchange subunits with each other or a Scar/AbiA/DdBrk1 subcomplex from *Dictyostelium*, we designed and purified DdBrk1 mutant M1C, in which the first methionine was replaced by cysteine, allowing labeling with Alexa 488 to obtain fluorescent DdBrk1_488. Additionally, we purified DdBrk1 as a fusion with maltose-binding protein (MBP-DdBrk1). The 43 kg/mol MBP moiety of the fusion protein drastically increased the hydrodynamic radius (r_H_) of the DdBrk1 trimer, which allowed us to monitor changes in the composition of the complex, after incubation with DdBrk1_488. After mixing of 100 nM DdBrk1_488 and 1.4 µM MBP-DdBrk1, and incubation for two hours on ice, the reaction mixture was subjected to gelfiltration during which DdBrk1_488 was detected by absorbance at 488 nm (Fig. 5A; compare blue and yellow lines), while elution of all proteins was simultaneously tracked by absorbance at 280 nm (Fig. 5B). While MBP-DdBrk1 was undetectable by absorbance measurement at 488 nm, it was clearly visible at 280 nm (Fig. 5A,B; black lines). Notably, a species with an elution volume similar to MBP-DdBrk1 appeared in the presence of DdBrk1_488 (Fig. 5A; blue line), demonstrating incorporation of labeled protein into a complex with a significantly increased r_H_ when compared to DdBrk1_488 alone (Fig. 5A, B; yellow line). The slightly smaller r_H_ of the MBP-DdBrk1/DdBrk1_488 complexes in comparison to MBP-DdBrk1 (Fig. 5B; black and blue lines) was caused by replacement of either one or two MBP-DdBrk1 subunits by DdBrk1_488, and excludes possible association of DdBrk1_488 with trimeric MBP-DdBrk1, which would have lead to an increase of r_H_. This experiment demonstrates dynamic exchange of subunits within DdBrk1 complexes at low micromolar concentrations, pointing out that homotrimeric DdBrk1 is not static, but rather dynamically exchanges subunits. Next, we coexpressed and purified GST-DdBrk1/Scar(1-225)/AbiA(1-149) from bacterial extracts by affinity chromatography. After removal of the GST-tag and subsequent purification the proteins were also incubated with 100 nM DdBrk1_488 and subjected to gelfiltration. The elution profile monitored by the absorbance at 488 nm clearly showed an increased r_H_ for DdBrk1_488 (Fig. 5A; red line), indicating the exchange of DdBrk1 by DdBrk1_488 in the Scar(1-225)/AbiA(1-149)/DdBrk1-complex (Fig. 5A,B; yellow and red lines). These findings suggested that the DdBrk1 trimer may be in a dynamic equilibrium with its monomeric form.

To test whether DdBrk1_488 trimers tend to dissociate at concentrations in the nanomolar range, 8 to 800 nM DdBrk1_488 were analyzed in sedimentation velocity experiments in an analytical ultracentrifuge monitoring the fluorescence of the Alexa 488 dye. At the highest concentrations examined, DdBrk1_488 sedimented slightly faster than the unlabelled protein (s_20,W_ = 2.3 S compared to 2.1 S for DdBrk1, Fig. 5C; red line) most likely due to the mass increase caused by the addition of the fluorescent dye. At lower protein concentrations this peak shifted to lower s-values and a second peak with an s_20,W_ of about 1.2 S appeared in the c(s) distributions (Fig. 5C). These results clearly show that DdBrk1_488 trimers dissociate at protein concentrations in the lower nanomolar range. The fact that the s-value of the faster sedimenting boundary decreases with decreasing protein concentration is an indication that the dissociation of the homotrimer is fast compared to the time scale of the sedimentation experiment. For a fast reaction the formation of a reaction boundary is expected in which a coupled sedimentation of the different oligomeric states takes place [44]. The s-value of this reaction boundary reflects the fractional time the molecule spends in the complex state and therefore depends on the total concentration of the protein. The species sedimenting with about 1.2 S most likely reflects monomeric DdBrk1_488 and comprises more than half of the total protein at a concentration of 8 nM, provided the quantum yield of DdBrk1_488 does not depend on its oligomeric state. To check whether the dynamics of the DdBrk1_488 homotrimer formation allows for subunit exchange between different variants of DdBrk1, a mixture of 1.4 µM MBP-DdBrk1 (see above) and 100 nM DdBrk1_488 was examined in a sedimentation velocity experiment monitoring the fluorescence of DdBrk1_488. In this reaction mixture most of the DdBrk1_488 sedimented faster than the homotrimer yielding a maximal s_20,W_ of about 6 S (data not shown). This clearly shows that even at a concentration of 100 nM DdBrk1_488 and in presence of 1.4 µM MBP-DdBrk1 subunits are able to swap between different complexes, which is in line with the results of the gelfiltration experiments described above.

### Conclusions

**Figure 6:**
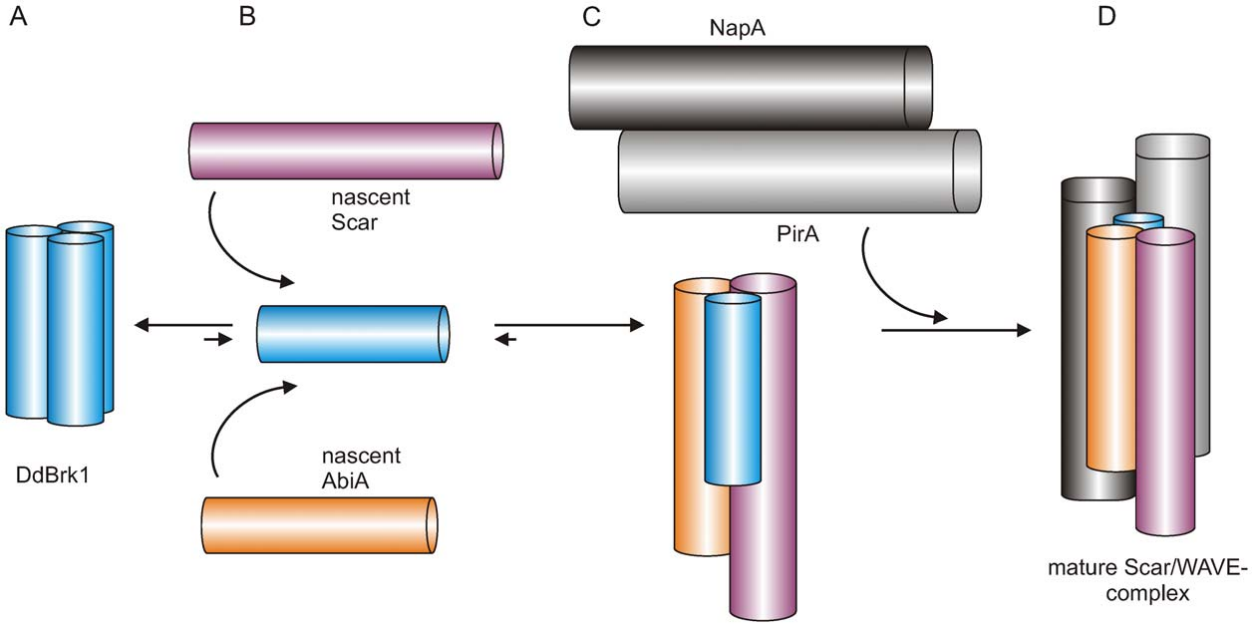
Proposed model for the assembly of the Scar/WAVE-complex. (A) Homotrimeric DdBrk1 maintains a dynamic pool of monomers, (B) which immediately associate with nascent Scar/WAVE and Abi proteins to form heterotrimeric subcomplexes. (C) Immediately thereafter, Scar/Abi/Brk1 heterotrimers are captured by the heterodimeric Nap/Pir platform to form the mature Scar/WAVE-complex (D).

Based on these results and previous findings, in particular the observed incorporation of exogenously added oligomeric human Brk1 into the mature Scar/WAVE-complex *in vivo* [26], we suggest that a constant pool of Brk1 homotrimers allows for formation of a given number of monomers, which directly bind and stabilize *de novo* synthesized Scar/WAVE and Abi subunits (Fig. 6A,B). Since the activation of the Arp2/3-complex is a highly regulated process *in vivo*, we assume that heterotrimeric Scar/AbiA/DdBrk1-complexes immediately associate with the heterodimeric Nap/Pir platform to mask the VCA domain of Scar/WAVE proteins to prevent the unregulated activation of the Arp2/3-complex (Fig. 6C,D). This is consistent with previous observations showing that stability of Nap and Pir proteins is apparently not affected in the absence of Scar/WAVE, Abi or Brk1 proteins *in vivo*, and by the fact that heterodimeric Nap/Pir remains stable after coexpression in insect cells [21], [23], [31], [32], [36]. Notably, Brk1 forms the majority of the surface contacts between heterotrimeric HsBrk1/Abi2/WAVE1 and Nap/Pir platform within the trimeric coiled-coil region (Fig. 4A,B). We therefore propose that in the final assembly step, Brk1 serves as an adapter protein to stabilize and coordinate the assembly of the heterotrimer and the binding to the Pir/Nap platform to assemble the mature Scar/WAVE-complex.

## Methods

### Expression construct

The coding sequences of all *Dictyostelium discoideum* proteins of this study were amplified from a λgt11-cDNA library. For DdBrk1 the following primers were used: DdBrk1_BU GCGGGATCCATGTCAACAAAAACAAATATTCAA and DdBrk1_SD CGCGTCGACTTATTCTTGTACAGTCTTGAATGT. The DdBrk1 M1C mutant was amplified using the primers DdBrk1_M1C_BU GCGGGATCCTGTTCAACAAAAACAAATATTCAAAA and DdBrk1_SD CGCGTCGACTTATTCTTGTACAGTCTTGAATGT. These primers contain *BamH*I and *Sal*I sites to facilitate cloning. The digested PCR fragments were inserted into the corresponding sites of the *E. coli* expression vectors pGEX-6P1 (GE-Healthcare) and pMal-c2x (New England Biolabs). Scar (aa1-225) and AbiA (aa1-149) fragments were amplified using the primers Scar_NcoU GCGCCATGGTATTAATTACAAGATATTTACCATC and Scar_*SD GCGGTCGACTTATGGACTTTCAATATTGATAGTAAT carrying *Nco*I and *Sal*I sites, and AbiA_BglII+1 GCGAGATCTCATGAGTGAATCAATCGATATTAACGTTTATT and AbiA_*XhoI GCGCTCGAGTTAAATACCATAAGAAATTGGTTTATG carrying *Bgl*II and *Xho*I sites for insertion into expression vector pRSF-Duet-1 (Merck). The coding sequence of GST-DdBrk1 was amplified from pGEX-6P1_DdBrk1 using the primers GST_RU+1 CGCGAATTCGATGTCCCCTATACTAGGTTA<1?show=[fo]?>TTG and DdBrk1_SD CGCGTCGACTTATTCTTGTACAGTCTTGAATGT carrying *EcoR*I and *Sal*I sites and inserted into the same site of pET-Duet-1 (Merck). All constructs were verified by sequencing.

### Protein purification

*D. discoideum* Brk1 and DdBrk1_M1C mutant were purified from *E. coli* host BL21 DE3 as N-terminally GST- or MBP-tagged fusion proteins. Briefly, the GST-fusion proteins were purified on glutathione sepharose (GE Healthcare), eluted with buffer A containing 20 mM Tris/HCl pH 7.3, 220 mM NaCl, 1 mM EDTA, 10% glycerol (v/v) supplemented with 30 mM reduced glutathione (Sigma) and subsequently cleaved with PreScission protease (GE Healthcare) at a molar ratio of 500∶1. The resulting DdBrk1 protein carries the extra amino-acids GPLGS at its N-terminus. After cleavage, the proteins were separated by anion exchange chromatography using a Mono Q 4.6/100 PE column (GE Healthcare) as the GST moiety remained in the flow through. After washing of the column with 2 column volumes of buffer A, bound DdBrk1 was eluted with buffer B, containing 20 mM Tris/HCl pH 7.3, 500 mM NaCl, 1 mM EDTA, 10% glycerol (v/v). The resulting DdBrk1 fraction was dialyzed against PBS containing 2.7 mM KCl, 1.8 mM KH2PO4, 10 mM Na2HPO4, 140 mM NaCl, pH 7.3, and was further purified by size-exclusion chromatography on a Superdex-S75 10/300 column (GE Healthcare) equilibrated with PBS. The protein was then dialyzed three times against PBS for analytical ultracentrifugation experiments or buffer C containing 20 mM Hepes pH 7.3, 50 mM NaCl, 1 mM DTT, 0.1 mM EDTA, 0.01% NaN3 for crystallization. MBP-DdBrk1 was purified using buffer D containing 20 mM Tris/HCl pH 8.0, 300 mM NaCl, 1 mM EDTA on amylose sepharose resin (New England Biolabs). The protein was eluted with buffer D supplemented with 30 mM maltose. The resulting MBP-DdBrk1 protein was subsequently dialyzed three times against PBS and further purified by size exclusion chromatography on a Superdex-S200 26/60 column (GE Healthcare) equilibrated with PBS using an Äkta purifier system (GE Healthcare).

Scar(aa1-225)/AbiA(aa1-149)/GST-DdBrk1 were coexpressed in *E. coli* host BL21 DE3 using pET-Duet_GST-DdBrk1 and pRSF-Duet_Scar(aa1-225)/AbiA(aa1-149) constructs and purified by glutathione sepharose affinity chromatography. After elution of GST-tagged DdBrk1 in complex with untagged Scar and Abi fragments as described above using PBS supplemented with 30 mM reduced glutathione (Sigma), the GST tag of DdBrk1 was cleaved with PreScission protease and removed by size-exclusion chromatography on a Superdex-S75 26/60 column (GE Healthcare) equilibrated with PBS. Purity of the samples was assessed by SDS-PAGE and Coomassie blue staining.

### Protein labeling

Purified DdBrk1-M1C was dialyzed three times against PBS and mixed with a 5-fold molar excess of Alexa-488 Fluor C5 maleimide (Invitrogen). The reaction was carried out in a degassed solution under protective nitrogen atmosphere on an end-to-end shaker for 3 h at 21°C. Free dye was removed by size exclusion chromatography on a Superdex-S75 10/300 column (GE Healthcare) equilibrated with PBS and subsequent dialysis. The degree of labeling was estimated by absorption spectrum measurement on a Jasco V-560 UV/VIS-spectrophotometer according to the instructions of the manufacturer (Invitrogen) using an extinction coefficient at 488 nm of the dye ε = 73,000 cm^−1^M^−1^.

### Generation of antibodies

Polyclonal antibodies were obtained by immunizing female white New Zealand rabbits with either recombinant GST-tagged DdBrk1 or two PirA peptides SGFEPAEAVPNKKSKEVEEKVQIPAR (aa 526–551), DKPYKTQLELAHFNGKLHTPKSRFD (aa 715–739) coupled to KLH (Thermo Scientific) together with complete Freund's adjuvant (Sigma) following standard procedures. Specificity of the anti-DdBrk1 antibodies was assessed by Western blotting comparing reactivity with MBP or MBP-fused to DdBrk1 (see Fig. 1B). Monoclonal anti-actin antibody 224–236-1 was previously described [45].

### Western blotting

For immunoblot analysis, total cellular proteins of *D. discoideum cells* were incubated in reducing SDS sample buffer and separated using 16% Tris-Tricin or 10% Tris-Glycin SDS-PAGE gels. After semi dry blotting onto PVDF membranes (GE-Healthcare), the membranes were blocked with 4% BSA (Sigma) in NCP containing 10 mM Tris/HCl pH 8.0, 150 mM NaCl and 0.05% Tween-20 for 20 min at room temperature (RT). After washing three times with NCP, the membranes were incubated at RT overnight with polyclonal antibodies (0.5 µg/ml in NCP). The membranes were subsequently washed three times in NCP and incubated for 2 h at RT with alkaline phosphatase-conjugated goat anti-rabbit antibodies (Dianova) diluted 1∶3,000 in NCP. After washing three times in NCP, the blots were developed using 5-bromo-4-chloro-3-indolyl phosphate (BCIP) (Carl Roth).

### Analytical Gelfiltration

Gelfiltration experiments were carried out on an Äkta Purifier HPLC-system (GE-Healthcare) using 500 µl sample volumes on a Superdex-S200 10/300 column (GE-Healthcare) at a flow rate of 0.5 ml/min at 4°C in PBS. Absorbance was measured with an UV-900 module (GE-Healthcare) at 280 nm and 488 nm oscillating with an averaging time of absorbance measurement of 1.28 s.

### Analytical ultracentrifugation

Sedimentation equilibrium experiments were carried out in a Beckman Optima XL-A analytical ultracentrifuge at 18,000 and 26,000 rpm in an An-60 Ti rotor at 10°C until no further change in absorbance could be detected for at least 12 h. Scans from these 12 h were averaged and molar mass determination was performed using a model of a single species, as described previously [46]. Standard 3 mm or 12 mm double sector centerpieces filled with 40 µl or 150 µl samples, respectively were used and absorbance was detected at 280 nm using the UV/Vis scanning optics of the centrifuge.

Sedimentation velocity experiments were performed at 50,000 rpm in a Beckman/Coulter ProteomeLab XL-I equipped with a fluorescence detection system (AU-FDS, Aviv Biomedical) at 20°C using an An-50 Ti rotor. Measurements using the absorbance optics of the centrifuge were carried out in 3 mm or 12 mm standard double sector cells filled with 100 µl or 400 µl samples, respectively and the signal was detected at 280 nm or 230 nm, depending on the protein concentration used. Concentration profiles of DdBrk1_488 were measured with an excitation wavelength of 488 nm and emission was detected through a pair of long pass (>505 nm) dichroic filters using special cell housings and standard 3 mm centerpieces as described previously [47]. Data were analyzed using the program package SEDFIT, which provides a model for diffusion corrected differential sedimentation coefficient distributions (c(s) distributions) [48]. Partial specific volume, buffer density and viscosity were determined by the program SEDNTERP [49] and were used to correct the experimental sedimentation coefficients to s_20,W_.

Concentration of unlabelled DdBrk1 was determined spectrophotometrically, using an absorption coefficient ε_280 nm_ = 6990 M^−1^ cm^−1^ calculated from amino acid composition [50], and is given in monomers throughout the text. All analytical ultracentrifugation experiments were carried out in PBS except for experiments designed to analyze potential changes of the multimerization state in the presence of Ca^2+^-ions. These experiments were performed in 20 mM Tris/HCl pH 8.0, 150 mM NaCl and varying concentrations of Ca^2+^ or EDTA.

### Crystallization and Structure Determination

Crystals were obtained in hanging drop geometry using 40% MPD, 0.2 M CaCl_2_ as reservoir solution. 1.5 µl of reservoir was added to an equal volume of DdBrk1 (16 mg/ml) and incubated at 20°C. Hexagonal rod-shaped crystals appeared within a few days. Crystals were backsoaked in mother liquor prior to flash freezing in liquid nitrogen. Data collection at the synchrotron was carried out at 100 K. Diffraction data were indexed, integrated and scaled using the XDS-package [51]. Identification of anomalous sites and phasing/density modification was done using the SHELXC/D/E-program package [40] with the HKL2MAP-interface [52]. High-resolution data allowed automatic model building using ARP/WARP [41]. The preliminary model was refined in cyclic rounds of manual model building in COOT [53] and refinement using phenix.refine [54]. The final model showed no outliers in the Ramachandran plot and reasonable R-factors and geometries. Atomic coordinates and structure factors were deposited in the Protein Data Bank with accession number 3PP5. The crystallographic parameters of the structure are listed in Table 1. All figures were prepared with PyMOL [55].
